# Protocol to dissect and dissociate the mouse brainstem for single-cell RNA-seq applications

**DOI:** 10.1016/j.xpro.2024.102908

**Published:** 2024-03-08

**Authors:** Wiktor S. Phillips, Naify Ramadan, Athina Samara, Eric Herlenius

**Affiliations:** 1 Department of Women’s and Children’s Health, Karolinska Institutet, Stockholm, Sweden; 2 Astrid Lindgren Children’s Hospital, Karolinska University Hospital, Stockholm, Sweden; 3 Department of Biomaterials, FUTURE, Center for Functional Tissue Reconstruction, University of Oslo, Oslo, Norway

**Keywords:** Cell isolation, Single Cell, Developmental biology, Neuroscience

## Abstract

Processing dissociated cells for transcriptomics is challenging when targeting small brain structures, like brainstem nuclei, where cell yield may be low. Here, we present a protocol for dissecting, dissociating, and cryopreserving mouse brainstem that allows asynchronous sample collection and downstream processing of cells obtained from brainstem tissue in neonatal mice. Although we demonstrate this protocol with the isolated preBötzinger complex and downstream SmartSeq3 cDNA library preparation, it could be readily adapted for other brainstem areas and library preparation approaches.

## Highlights

• Steps for taking transverse slices of the medulla oblongata that contain the preBötC
• Detailed punchout procedure for preBötC single-cell dissociation
• Asynchronous collection, fixation, and methanol cryopreservation
• Optimized for SmartSeq3 cDNA library preparation and FACS

## Abstract (editor highlights)

Publisher’s note: Undertaking any experimental protocol requires adherence to local institutional guidelines for laboratory safety and ethics.

## Graphical abstract

**Figure:**
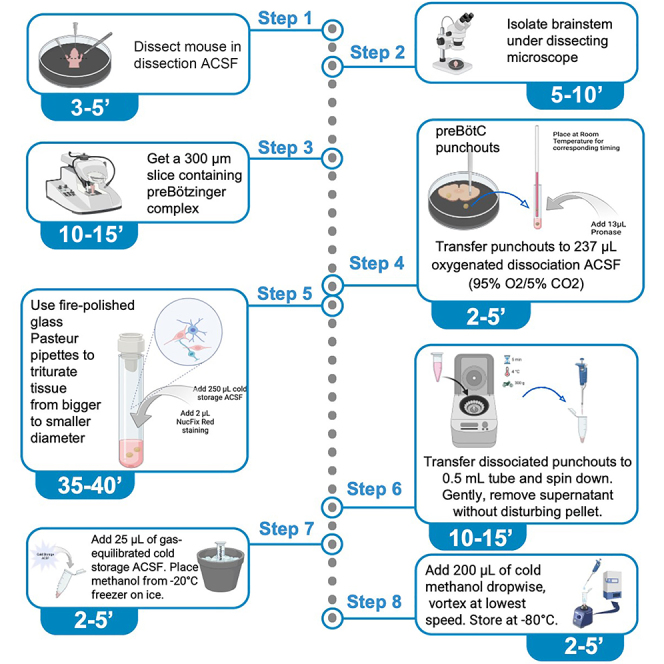

## Before you begin

The protocol below describes specific steps for microdissecting tissue punchouts of the preBötC but could be adapted for other brainstem regions by cutting tissue slices from different locations along the rostrocaudal extent of the brainstem and targeting other regions of interest when taking punchouts.

### Fire-polishing glass Pasteur pipettes

**Timing: 30–60 min**

1. Hold the tip of a glass Pasteur pipette over the flame of a gas burner for approximately 1–3 s.
2. Measure the tip diameter using the optics and measurement reticle of a microforge or benchtop microscope.
3. Repeat steps 1–2 as necessary to progressively shrink the pipette tip to the desired diameter.
4. Color code the fire-polished pipettes by size using colored vinyl tape according to the sizing table (Table 1).

**Table 1:** Pipette color coding and polished tip inner diameter

Label color	Tip inner diameter (μm)
Black	800–1000+
Green	600–800
Yellow	250–600
Red	150–250

### Silanization of dissociation glassware

**Timing: 70–100 min**

5. Silanize dissociation glassware.
  a. Pasteur pipettes.
    i. Aliquot approximately 1.5–2.0 mL of Sigmacote into a 1.5–2.0 mL microtube.
    ii. Immerse the glass pipette into the Sigmacote and pipette up and down 3 times.
    iii. Ensure that both interior and exterior surfaces of the pipette are exposed to the reagent.
    iv. Repeat as necessary for additional pipettes. The Sigmacote in the microtube may be reused to coat additional glass pipettes.
  b. Glass centrifuge tubes.
    i. Use a transfer pipette to fill a glass conical tube with approximately 2.0 mL of Sigmacote.
    ii. Immediately remove the Sigmacote.
    iii. Repeat as necessary for additional glass tubes.
    iv. You may reuse the same volume of Sigmacote, transferring it to additional glass tubes to coat multiple tubes in series.
6. Allow coated glassware to air dry on a laminar air flow bench or underneath a fume hood.
7. After the heptane solvent evaporates (approximately 30–60 min), bake the glassware in the oven at 100°C for 30 min.

***Alternatives:*** Silanization helps minimize cell loss during trituration and dissociation. This section can be skipped if cell yield is otherwise acceptable (See troubleshooting).

***Note:*** Silanization of the Pasteur pipettes and conical glass centrifuge tubes only needs to be performed once, with occasional recoating as necessary. Once coated and baked, silanized glassware can typically be reused for 2 weeks before needing to be recoated (See troubleshooting).

### Fabricating Sylgard-lined petri dishes

**Timing: 15 min + 1–2 days of curing**

8. Mix Sylgard elastomer and curing agent in a 50 mL centrifuge tube using a metal spatula according to the manufacturer’s instructions.

***Note:*** A total volume of 30–50 mL is sufficient to coat the bottom of a 100 mm diameter Petri dish.

9. Incrementally add carbon black to the Sylgard and mix using a spatula, stopping when the elastomer mixture is completely opaque.

***Note:*** The total amount of carbon black varies depending on the volume of Sylgard to be pigmented. Usually, less than 1 g is sufficient to blacken the elastomer.

10. Pour the blackened Sylgard mixture into a Petri dish, evenly dispersed to a depth of 3–5 mm from the bottom of the dish.
  a. For fine dissection dishes, pour into the bottom of a 100 mm × 20 mm glass Petri dish.
  b. For gross dissection plates, pour into the lid of a 150 mm diameter plastic petri dish.
11. Cover the Sylgard-filled dish with a lid.
  a. Cure undisturbed for at least 48 h at 20°C–24°C or until the Sylgard has solidified.

***Note:*** The cured elastomer should be free from bubbles, which slowly rise out of the mixture at room temperature.

**CRITICAL:** Sylgard cures more quickly at elevated temperatures (e.g., >40°C; see manufacturer instructions). However, heated curing may trap air bubbles in the Sylgard, producing an uneven surface. In addition, lower temperature curing results in a softer elastomer that is easier to pierce with pins during dissections.

***Note:*** Sylgard-lined dishes should be washed with dish soap or a laboratory detergent and rinsed with ethanol each day after dissections (See cleaning reusable dissection dishes). It is not critical that they be completely sterilized (e.g., via baking).

### Cleaning reusable dissection dishes

**Timing: 10–15 min**

12. Clean all Sylgard-lined dissection dishes thoroughly with detergent.
  a. Wash with a solution of 1% w/v Alconox detergent in deionized water.
  b. Thoroughly rinse dissection dishes, first with deionized water and then 70% ethanol.
  c. Allow the dissection dishes to air dry before use.

### Cleaning dissociation glassware

**Timing: 3 h**

Dissociation glassware include conical bottom glass centrifuge tubes and fire-polished Pasteur pipettes used during cell trituration steps (see dissociation of brainstem tissue punches steps 46-52).

13. Prepare glass cleaning solution by dissolving 10 g of Alconox into 1 L of purified (e.g., MilliQ) water.
14. Add the cleaning solution to an ultrasonic bath up to the manufacturer-marked fill line.
15. Set the temperature of the ultrasonic bath to 50°C and degas the solution for 30 min prior to use.
16. Place the glassware into the cleaning solution, tipping or rotating the glass as necessary to free bubbles from the submerged tubes and pipettes.
17. Sonicate the glassware for 30 min.
18. Remove the glassware from the sonicator and rinse thoroughly with deionized water.
19. Wrap all the glassware pieces bundled together in a sheet of aluminum foil.

***Alternatives:*** Lay all the pieces in a stainless-steel tray and cover the glassware with a tray lid, or a piece of aluminum foil.

20. Post-processing.
  a. If preparing glassware for silanization, dry by heating in the oven at a moderate temperature (60°C–80°C) for 2 h.
  b. If preparing glassware for sample preparation, heat sterilize the glass by baking in an oven at 160°C for 2 h, or 170°C for 1 h.
21. Store the clean and dried glassware covered in a tray or wrapped in aluminum foil on the bench of a biosafety cabinet.

**CRITICAL:** After sonication, handle glassware with gloves and rinse thoroughly with deionized water in order to remove all detergent residue.

**Pause point:** Sterilized glassware can be stored wrapped in foil inside the biosafety cabinet until needed.

### Preparation of 10× pronase stock

**Timing: 10 min**

22. In a 50 mL conical tube, dissolve 1 g of dehydrated pronase in purified water up to a final volume of 50 mL.

***Note:*** Dehydrated pronase should be reconstituted in sterile deionized water, at a concentration of 20 mg per mL.

**CRITICAL:** Minimize foaming during reconstitution. Do not vigorously shake the solution. Instead, encourage dissolution by gently rocking and inverting the (capped) tube.

23. Aliquot the dissolved pronase stock into 1.5 or 2.0 mL microtubes and store at −20°C until use. Pronase stocks may be stored for a maximum of 1 month.

**CRITICAL:** Pronase degrades at room temperature. After reconstitution in water, immediately aliquot the stock solution and transfer to a –20°C freezer without pausing. Frozen aliquots of pronase may be stored for up to 1 month at –20°C before use. Upon thawing individual aliquots, keep the microtube of pronase on ice (or cooled at 4°C) and use in downstream protocol steps within 2 h. Do not refreeze aliquots after thawing; discard excess thawed volume after 2 h.

***Note:*** Pronase is used because it allows dissociation to be performed without heating the solution, simplifying the dissociation step. Dissociation at lower temperatures (e.g. 20–24°C) also helps reduce technical noise in downstream transcriptomics applications.^1^

### Preparation of nuclear stain stocks

**Timing: 10–20 min per stock**

24. Live-or-Dye NucFix Red dye.
  a. Prepare a 1000× dye stock solution in DMSO according to the manufacturer’s instructions.
  b. Store at –20°C protected from light. Stocks can be used for up to 1 month after freezing.
  c. Per the manufacturer’s recommendation, minimize the number of freeze thaw cycles.
25. Hoechst 33342 dye (5 mg/mL).
  a. If working with Hoechst in solid form, you can dissolve the dye at 5 mg/mL in DMSO.
    ***Note:*** Vortexing, sonication, and warming (up to 50°C) may be necessary to fully dissolve the Hoechst solid.
  b. Dispense the stock solution of Hoechst dye into 0.05–0.5 mL aliquots and store at −20°C protected from light until use.
  c. Hoechst stocks can be used for 1–3 months after freezing.
    ***Alternatives:*** Hoechst 33342 can be substituted with Hoechst 34580.
    **CRITICAL:** Unlike propidium iodide, NucFix Red binds covalently to DNA, and is thus robust to methanol fixation and washing with SSC buffer during rehydration. We therefore do not recommend substitution with another viability stain.

### Preparation of dissection artificial cerebrospinal fluid (ACSF)

**Timing: 25 min**

26. Dissolve all reagents (except the divalent salts: CaCl_2_ and MgSO_4_) into a final volume of ≤990 mL purified water.
27. Equilibrate the solution by bubbling with 95% O_2_ / 5% CO_2_ gas for at least 15 min.
28. Slowly add the divalent salts to the solution while stirring then top off to a final volume of 1000 mL using deionized H_2_O.
29. Run each solution through a 0.22 μm bottle-top filter into sterilized 1 L glass bottles.
30. Seal, label, and store each solution at 4°C until ready for use.
31. Dissection ACSF may be stored at 4°C for up to 1 week.

### Preparation of Base Solution for dissociation ACSF & cold storage ACSF solutions

**Timing: 15 min**

32. Dissolve the reagents listed in Table 2 (excluding the divalent salts; CaCl_2_ and MgSO_4_) into a volume of 170 mL deionized pure water.

**Table:** Table 2. Base solution for Dissociation ACSF & Cold Storage ACSF

Reagent	Final concentration	Stock concentration	Amount per 1 L	Amount per 200 mL
KCl	2.5 mM	1 M	2.5 mL	500 μL
NaH_2_PO_4_	1.25 mM	1 M	1.25 mL	250 μL
NaHCO_3_	30 mM	Solid	2.52 g	0.5 g
D-Glucose	25 mM	Solid	4.5 g	0.9 g
Sodium Ascorbate	5 mM	Solid	0.99 g	198 mg
N-acetyl-L-cysteine	12 mM	0.5 M	24 mL	4.8 mL
Ethyl Pyruvate	3 mM	Liquid	0.33 mL	66 μL
*myo*-Inositol	5 mM	Solid	0.54 g	108 mg

**Divalent Cations**				

CaCl_2_	0.5 mM	1 M	0.5 mL	100 μL
MgSO_4_	10 mM	1 M	10 mL	2 mL

Use immediately for making Dissociation ACSF and/or Cold Storage ACSF.
33. Equilibrate the solution by bubbling with 95% O_2_/5% CO_2_ gas for at least 10 min.
34. While continuously stirring the solution, slowly add the divalent cation salts to the equilibrated solution then top off the solution with additional water to a final volume of 180 mL.
35. Divide the Base Solution equally (2 × 90 mL) into two separate 100 mL graduated cylinders.
36. Proceed directly to the preparation of Dissociation ACSF and Cold Storage ACSF.

### Preparation of dissociation ACSF solution

**Timing: 5–10 min**

37. Dissolve the reagents listed in Table 3 into a 90 mL volume of Base Solution.

**Table:** Table 3. Dissociation ACSF—Additional reagents

Reagent	Final concentration	Stock concentration	Amount per 100 mL
HEPES (pH 7.4 at 21°C)	20 mM	1 M	2 mL
Phenol red	0.001%	0.5%	200 μL
Trehalose dihydrate	132 mM	Solid	5 g

Store finished Dissociation ACSF at 4°C for up to 1 week.
38. Top off the solution to 100 mL with additional purified water and sterilize by filtering the solution through a 0.22 μm bottle-top filter into a 100–250 mL sterile bottle.
39. Seal the bottle and store at 4°C for no more than 1 week.

### Preparation of cold storage ACSF solution

**Timing: 5–10 min**

40. Dissolve the reagents listed in Table 4 into a 90 mL volume of Base Solution.

**Table:** Table 4. Cold Storage ACSF—Additional reagents

Reagent	Final concentration	Stock concentration	Amount per 100 mL
HEPES (pH 7.4 at 4°C)	20 mM	1 M	2 mL
Trehalose dihydrate	132 mM	Solid	5 g

Store finished Cold Storage ACSF at 4°C for up to 1 week.
41. Top off the solution to 100 mL with additional purified water and sterilize by filtering through a 0.22 μm bottle-top filter into a 100–250 mL sterile bottle.
42. Seal the bottle and store at 4°C for no more than 1 week.

### Preparation of BSA stock

**Timing: 5–10 min**

43. Prepare a 1% w/v stock of BSA solution by rehydrating 1 g of dry lyophilized BSA in 100 mL total volume of PBS. Store at 4°C for a maximum of 1 month.

### Preparation of Resuspension Buffer

**Timing: 5–10 min**

44. Prepare 5 mL of Resuspension Buffer by first adding 0.75 mL of 20× SSC to a sterile 15 mL tube.
45. Add 200 μL of 1% stock BSA in PBS to the tube.
46. Add 50 μL of DTT (stock concentration 100 mM) to the tube.
47. Add 25 μL of Recombinant RNase inhibitor (stock concentration 40 U/μL) to the tube.
48. Top off the total volume to 5 mL with RNase- and DNase-free water. Store the finished buffer at 4C or on ice and use within 3 h for rehydration of methanol fixed samples (Steps 65–72).

### Institutional permissions

All experiments on mice must be performed in accordance with relevant institutional and national guidelines and regulations, and users should acquire permissions from their relevant institutions. In this study, the animals were reared and kept at the Department of Comparative Medicine, Karolinska Institutet, Stockholm, Sweden. The Stockholm Animal Research Ethics Committee granted permission for all animal experiments in this study (approval no. 15819-2017). The protocol is in accordance with European Community Guidelines and approved by the Regional Ethics Committee.

## Key resources table

**Table:** 

REAGENT or RESOURCE	SOURCE	IDENTIFIER
**Chemicals, peptides, and recombinant proteins**		

Calcium chloride	Merck/Sigma-Aldrich	442909
Ethyl pyruvate	Merck/Sigma-Aldrich	E47808
Lyophilized bovine serum albumin	Merck/Sigma-Aldrich	10735078001
Dithiothreitol (100 mM)	Promega	P1171
D-glucose	VWR	97061-166
Glycerol	VWR	4388.320
HEPES acid	Fischer Scientific	BP310-100
Hoechst 33342	Thermo Fisher Scientific	11544876
Magnesium sulfate heptahydrate	Merck/Sigma-Aldrich	M1880
Methanol	Merck/Sigma-Aldrich	322415-250ML
*myo*-Inositol	Merck/Sigma-Aldrich	57570
N-acetyl-L-cysteine	Merck/Sigma-Aldrich	1124220025
Phenol red	Merck/Sigma-Aldrich	P0290
Potassium chloride	VWR	60136-250ML
Pronase (PRON-RO)	Merck/Sigma-Aldrich	10165921001
RNasin Plus (for dissociation)	Promega	N2615
Recombinant RNase inhibitor (for rehydration buffer)	Takara	2313B
Sigmacote	Merck/Sigma-Aldrich	SL2-25ML
Sodium ascorbate	Merck/Sigma-Aldrich	11140-250G
Sodium bicarbonate	VWR	27776.296
Sodium chloride	VWR	BDH9286-500G
Sodium phosphate monobasic dihydrate	Merck/Sigma-Aldrich	71505
Standard sodium citrate (SSC) 10×	VWR	75801-018
Sylgard 184	Dow Corning	1673921
Trehalose dihydrate	VWR	28719.290
Trypan blue	VWR	97063-702
DMSO	VWR	N182-5X10ML
Alconox detergent	Merck/Sigma-Aldrich	Z273228
Terg-a-zyme enzyme detergent	Merck/Sigma-Aldrich	Z742918

**Critical commercial assays**		

Live-or-Dye NucFix Red	Biotium	32010

**Experimental models: Organisms/strains**		

Neonatal, male and female, mouse pups	Charles River	Crl:CD1(ICR)

**Other**		

Chemical fume hood or laminar airflow bench	Any	N/A
Laboratory gas burner	INTEGRA Biosciences	Fireboy
Microforge (or any microscope with a measuring scale)	Narishige	MF-830
Vibrating microtome	Campden	7000smz-2
Laboratory oven	Any	N/A
Heated ultrasonic water bath	Branson	CPX3800H
Digital timer	Any	N/A
Benchtop magnetic stirrer	Any	N/A
Benchtop vortex mixer	Scientific Industries	Vortex Mixer 2
Benchtop pH meter	Mettler Toledo	30671556
Flow cytometer	Sony	SH-800S
Sorting chip-100 um nozzle for SH800	Sony	LE-C3210
Variable volume manual pipettors (20 μL, 200 μL, 1000 μL)	Rainin	30456871
Analytical balance	Any	N/A
Dissociation glassware—DURAN centrifuge tube, conical bottom 30°, 25 mL, Ø 16 × 100 mm	DWK	242630901
Dissociation glassware—Glass Pasteur pipettes	VWR	14673-010
Crushed ice	Any	N/A
Aluminum foil	Any	N/A
Colored vinyl tape in colors: black, green, yellow, red.	3M	165GR4A
1000 μL Flowmi cell strainer 40 μm porosity	Bel-Art	H13680-0040
Qualitative filter paper (70 mm diameter circles; grade 3)	Munktell	110064
Sterile bottle-top filters PES 0.22 μm 1000 mL	VWR	514-0342
Sterile bottle filtration units PES 0.22 μm 150 mL	VWR	514-0328
35 mm sterile Petri dishes	Falcon	391-1998
100 mm × 20 mm borosilicate glass Petri dishes	VWR	391-0579
150 mm diameter Petri dish (only lids required)	Falcon	351058
50 mL disposable centrifuge tubes	VWR	10026-078
Stainless steel instrument tray	VWR	10196-164
Latex bulbs for pipettes	VWR	82024-550
LoBind protein 0.5 mL microcentrifuge tubes	Eppendorf	0030108434
1.5 mL microcentrifuge tubes	Eppendorf	0030123611
NIH style neuro punch ID 0.69 mm, OD 1 mm, 4.5 cm	AgnThos	18036-19
Carbon black	Claessons Trätjära AB	1007013
Scalpel blades	Swann-Morton	233-5472
Round/flat spatulas	VWR	82027-494
Dissection microknife	Fine Science Tools	10055-12
Angled dissection microscissors	Fine Science Tools	15008-08
Curved dissection microscissors	Fine Science Tools	15015-11
Fine forceps	Fine Science Tools	11254-20
Tissue forceps	Fine Science Tools	11021-12
Minutien pins	Fine Science Tools	26002-15
Syringe needles 27G	VWR	76582-392
Syringe needles 20G	VWR	76582-368
Gauze sponges (2 in × 2 in)	VWR	82004-740
20 μL sterile pipette tips	Rainin	17005860
200 μL sterile pipette tips	Rainin	30389240
1000 μL sterile pipette tips	Rainin	30389213
95% O2 – 5% CO_2_ gas tank	Any	N/A
1-hole Rubber stoppers	VWR	KART3823
1/16″ ID laboratory tubing	Tygon	ACF00003
Blunted syringe needles	Becton Dickson	305181
1/16″ barbed luer fittings	Cole-Parmer	EW-12028-47
1-way stopcocks	Braun	455980
2-way stopcocks	Braun	456020

## Materials and equipment

**Detergent Alternatives:** The protocol describes the procedure using the commercial detergent Alconox, but we have also reproduced it using Tergazyme, a similar detergent. Other laboratory-grade glassware detergents or solvents may also be adequate but have not been tested.

**Flow Cytometry Alternatives:** We have suggested a flow cytometer (Sony SH800S), but any comparable flow cytometer that can sort cells into well plates can be substituted.

**Table:** Dissection ACSF

Reagent	Final concentration	Stock concentration	Amount per 1 L	Amount per 200 mL
KCl	2.5 mM	1 M	2.5 mL	500 μL
NaH_2_PO_4_	1.25 mM	1 M	1.25 mL	250 μL
NaHCO_3_	30 mM	Solid	2.52 g	0.5 g
D-Glucose	25 mM	Solid	4.5 g	0.9 g
Sodium Ascorbate	5 mM	Solid	0.99 g	198 mg
N-acetyl-L-cysteine	12 mM	0.5 M	24 mL	4.8 mL
Ethyl-Pyruvate	3 mM	Liquid	0.33 mL	66 μL
*myo*-Inositol	5 mM	Solid	0.54 g	108 mg

**Divalent Cations**				

CaCl_2_	0.5 mM	1 M	0.5 mL	100 μL
MgSO_4_	10 mM	1 M	10 mL	2 mL

Store at 4°C for up to 1 week

**Table:** Resuspension Buffer

Reagents	Final concentration
20× SSC	3×
BSA (1% in PBS)	0.04%
DTT (100 mM)	1 mM
RNase Inhibitor	0.2 U/μL

Store on ice or at 4°C and use within 3 h for cell rehydration (Steps 65–72).

### Dissociation glassware

• DURAN Centrifuge Tube, conical bottom 30°, 25 mL, Ø 16 × 100 mm.
• Glass Pasteur pipettes (fire polished).

### Gas equilibration manifold build materials

• A manifold for controlled delivery of 95% O_2_ / 5% CO2 gas to multiple glass centrifuge tubes can be assembled *ad hoc* using the following materials:
  ○ 1-hole rubber stoppers.
  ○ 1/16″ ID laboratory tubing.
  ○ Blunted syringe needles.
  ○ 1/16″ barbed luer fittings.
  ○ 1-way stopcocks.
  ○ 2-way stopcocks.

***Note:*** For an example of an assembled manifold, see Figure S1.

## Step-by-step method details

### Gross dissection of neonatal mice

**Timing: 15–20 min**

This section describes the preliminary dissection procedure to remove the limbs, viscera, and skin of the mouse before extraction of the brainstem.

**CRITICAL:** From the beginning of the dissection, and throughout the rest of the protocol, practice standard aseptic techniques (i.e., wear gloves and lab coats, use 70% ethanol liberally to disinfect surfaces).

**CRITICAL:** Dissection instruments, such as glass droppers, scalpels, scissors, and tissue punches, should be heat sterilized in a glass bead sterilizer before each dissection.

**CRITICAL:** For best results, the gross dissection, fine dissection, and brainstem slicing (i.e. Steps 1–45) should be completed in less than 30 min. With practice, it can be done in less than 20 min.

1. Anesthetize a neonatal (P0-P7) mouse pup via isoflurane in an induction chamber.
2. Verify complete anesthesia via pinch response before proceeding.
3. Place the pup prone in the middle of a sterile cotton gauze pad laid on top of a Sylgard-lined gross dissection plate.
4. Use 27G syringe needles to pin both of the front paws outstretched laterally.
5. Place a third needle through the snout, rostral of the oculars.

***Note:*** All pins should be angled approximately 45 degrees outward to provide ample access and added stability.

**Figure 1:**
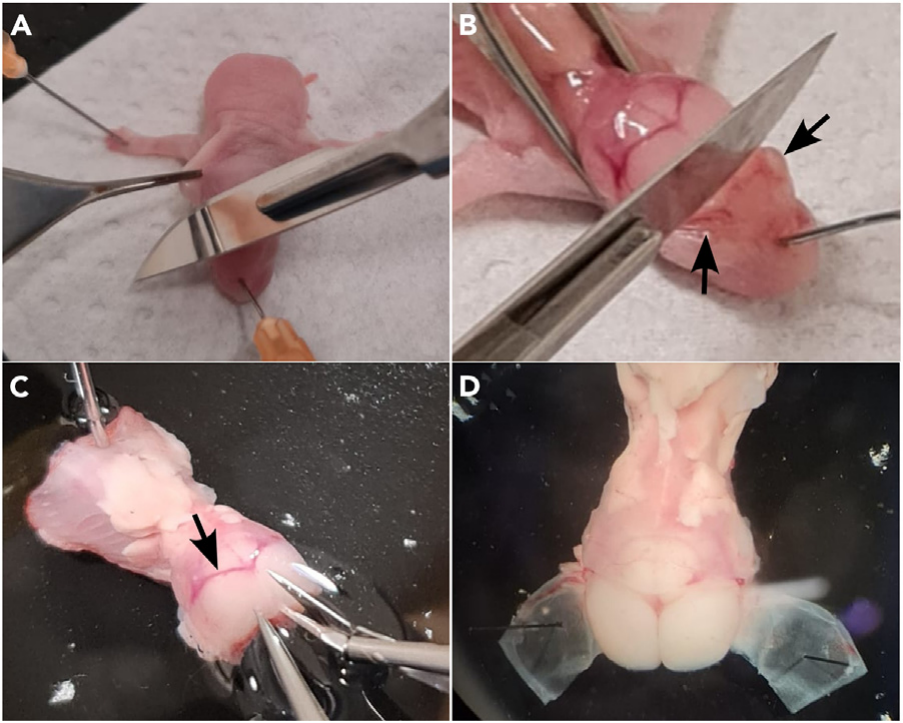
Gross dissection of the neonatal mouse (A) Forceps and scalpel are used to remove the skin on a euthanized mouse pup that has been pinned to a Sylgard plate through the snout and forelimbs. (B) Decerebration of the mouse pup via transection just caudal to the oculars. Arrows indicate location of oculars. (C) Example of making “flaps” from the skull by cutting along the suture lines. Arrows indicate location of lambdoid suture. (D) Example of finished flaps with insect pins anchoring the prep to the Sylgard base of the Petri dish.
7. Sever the frontal cortex by cutting with a gentle saw-blade movement in the coronal place, just caudal of the oculars.

***Note:*** It is important not to apply unnecessary pressure/force, but let the blade do the cutting (Figure 1B).

6. Using mouse-tooth forceps and a scalpel blade, remove the skin on the dorsal sides of the skull.
  a. Start from the oculars and continue to the middle of the abdomen (Figure 1A).

8. Sever away the hindquarters by performing a transection in the middle of the abdomen, just caudal of the rib cage, using a sawblade-like motion.
  a. Use mouse-tooth forceps to eviscerate the animal, leaving an empty abdominal cavity.
9. Using curved scissors, remove the front limbs by cutting away connective tissue between the abdomen and scapulae.
10. Transfer the reduced mouse carcass to a Sylgard-lined 100 mm Petri dish for fine dissection.

### Fine dissection of the brainstem and tissue mounting

**Timing: 15–20 min**

This section describes the procedure for extracting the brainstem and mounting it to the chuck of a vibrating microtome for calibrated tissue slicing.

***Note:*** Unless otherwise indicated, dissection ACSF should be prechilled to 0°C–4°C (e.g. on ice) and equilibrated via bubbling 95% O_2_/5% CO2 gas into the solution for at least 30 min before use.

11. Insert a 20G syringe needle through the abdomen.
  a. The body should be lying dorsal side up.

***Note:*** The needle should pierce the body adjacent to the spinal column on the rostral end of the remaining abdominal cavity and exit through the sternum on the ventral side, anchoring the body to the Sylgard base.

12. Using angled scissors, cut the skull along the midline.
  a. Start from the rostral end, following the sagittal suture until reaching the lambdoid (Figure 1C).
13. Make two additional scissor cuts in the skull.
  a. Cut laterally following the lambdoid sutures on the left and right, respectively, until approximately reaching the median horizontal plane.
14. With a pair of fine tip forceps, gently lift away the two "flaps" of skull from the dorsal surface of the brain.
  a. Use a minutien pin to pierce through each skull flap and into the Sylgard base of the dissection dish (Figure 1D).

***Note:*** Pins should be angled ∼45 degrees outward and placed 3–5 mm rostrolaterally away from the remaining skull without tearing off the skull flap. This provides (in total) three points of stability via pinning for subsequent dissection steps.

**Figure 2:**
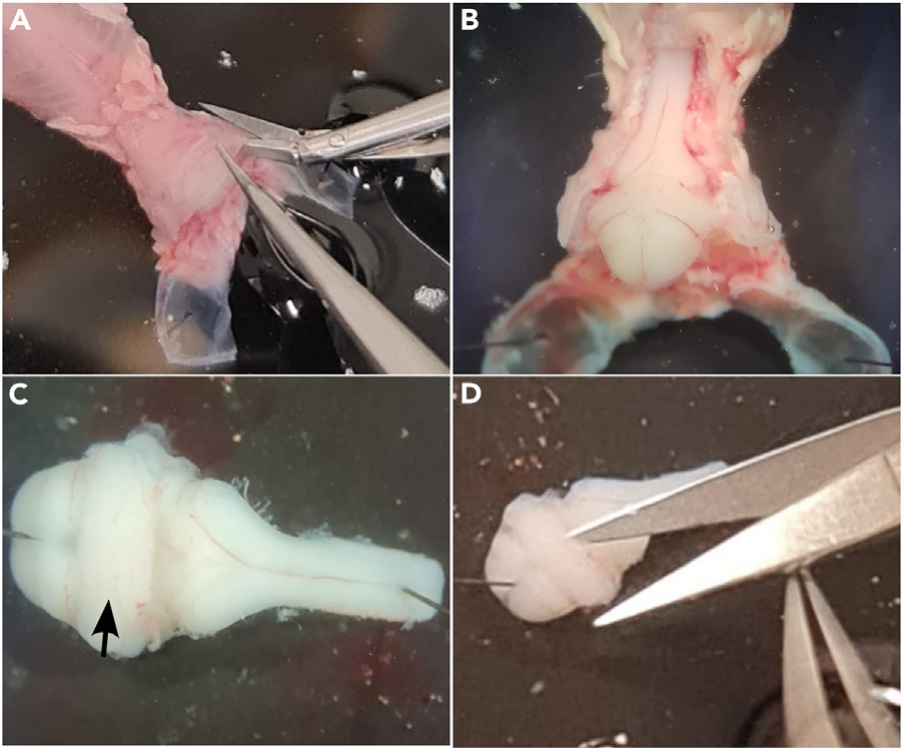
Fine dissection of the neonatal medulla and spinal cord (A) Spinal laminectomy is performed by cutting along the lateral regions of the remaining skull and vertebral bone, exposing the brainstem and spinal cord. (B) Example of exposed brainstem and spinal cord after removing the dorsal skull and vertebral bone. (C) The brainstem and spinal cord removed from the skull and pinned to the Sylgard dish. Arrows indicate cerebellum. (D) The cerebellum is removed with curved scissors, being careful to not cut the medulla.

***Note:*** Care must be taken not to pierce or sever the underlying brain structures. This is best achieved by cutting 2–3 mm at a time, alternating from left to right sides, and gently scraping off the meninges with the scissor blades before lifting away the detached bone.

***Note:*** The dorsal brainstem and cervical spinal cord should now be exposed.

15. Using fine tissue forceps and curved scissors, lift and cut away any excess fat and connective tissue remaining on the dorsal regions of the skull and spine, cleanly exposing the bone surface.
16. Using curved scissors or a scalpel blade, sever the exposed brain with a coronal cut at the level of the lambdoid.
  a. Remove the transected cortex/midbrain.
17. Perform a dorsal laminectomy by cutting the skull along the lateral edges of the brain/spinal cavity.
  a. Continue cutting from rostral to caudal, stopping only after exposing the full length of the cervical plexus (Figure 2A).

18. Use a pair of forceps to gently lift the brainstem.
  a. Cut away cranial and spinal nerves on the ventral side of the brainstem with angled scissors (Figure 2B).
19. Use scissors to transect the spinal cord caudal to the cervical plexus.
  ***Note:*** The brainstem and spinal cord should now be freed.
  a. Gently lift the brainstem-spinal cord out of the skull/spinal cavity.
  b. Discard the remaining carcass.
20. Lay the brainstem-spinal cord dorsal side up.
  a. Put a minutien pin through the caudal tip of the spinal cord into the Sylgard base.
  b. Put a second minutien pin through the tissue rostral to the cerebellum (Figure 2C).
21. Using curved scissors, carefully cut away and discard the cerebellum (Figure 2D).
  a. Lift away the choroid plexus (if any) from the 4^th^ ventricle using fine tipped forceps.

***Note:*** Be extra careful not to puncture or cut the medulla.

22. Remove the insect pins from the brainstem-spinal cord.
23. Using a clean razor blade and a cutting angle guide, make a right triangle-shaped piece of agar, with a 20-degree angle (20-70-90 degree triangle; see Figure 3A).

**Figure 3:**
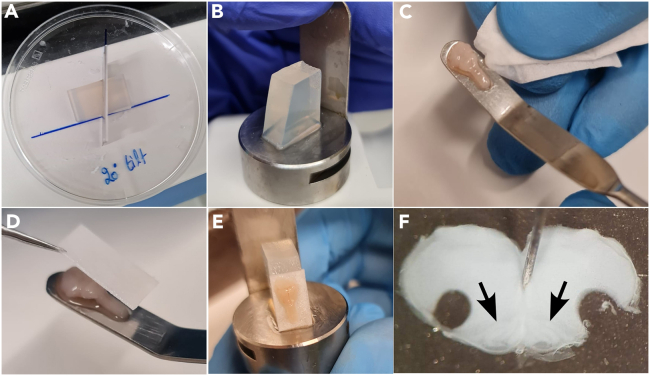
Mounting the dissected brainstem and example slicing results (A) A block of agar is precisely cut at a 20-degree angle as a substrate for supporting the preparation during slicing. (B) The angled agar block is fixed to the chuck of a vibrating microtome with cyanoacrylate glue. (C) The brainstem is placed onto a spatula, dorsal side facing upward, and excess ACSF is wicked away with the corner of a clean lint-free wipe. (D) A small piece of filter paper, saturated with cyanoacrylate glue, is gently placed onto the dorsal surface of the brainstem. (E) The filter paper (with attached brainstem) is then glued to the angled surface of the agar block such that the rostral end is facing upward. (F) A finished slice after punching out the bilateral regions containing the preBötC. Arrows indicate location of inferior olive.
24. Affix the short side of the block to the surface of the metal cutting chuck using cyanoacrylate glue.
  a. Place the prepared cutting block assembly aside (Figure 3B).
25. Apply 12 μL of cyanoacrylate glue to a ∼10 × 8 mm piece of filter paper, allowing the glue to soak into the fibers.
  a. Gently lay the glue-soaked filter paper on the lid of a Petri dish.
26. Using a large bent spatula, lift the brainstem-spinal cord out of the ACSF solution, with the dorsal side facing upwards.
  a. Wick away any excess moisture with a clean lint-free wipe without directly touching the tissue (Figure 3C).
27. Retrieve the glue-soaked filter paper with a pair of forceps.
  a. Gently apply the filter paper directly to the dorsal surface of the brainstem-spinal cord (Figure 3D).
28. Immediately immerse the glued brainstem-spinal cord back into solution.
29. Apply 12 μL of cyanoacrylate glue to the angled surface of the agar cutting block.
30. Remove the paper-affixed brainstem from the ACSF solution.
  a. Wick away excess solution with a lint-free wipe.
  b. Place the paper-affixed brainstem on the glued surface of the agar block (Figure 3E).
31. Firmly press the edges of the filter paper with a blunt probe or forceps to ensure that a strong bond is formed with the agar.
32. Immediately mount the cutting block into the bath of the vibrating microtome.

### Calibrated brainstem slicing and isolation of the preBötC

**Timing: 15–20 min**

This section describes how to take transverse slices of the medulla that contain the preBötC. These slices are further reduced by using a tissue punch to isolate the preBötC before dissociating the cells in later steps.

33. Fill the bath of the vibrating microtome with fresh gas-equilibrated dissection ACSF.
34. Ensure the angled surface of the agar block is positioned perpendicular to the vibrating microtome cutting blade.
35. Advance the blade, stopping before touching the brain tissue.
  a. Manually adjust the blade position until the cutting edge lies approximately 1 mm in front of the dorsal border of brainstem and 1 mm below the rostral border of the tissue.
36. Take a single cut of the brainstem at an advance speed of 100–200 μm/s.
37. Proceed to cut serial sections at a rate of 100–150 μm/s and 400 μm thickness until the opening of the 4^th^ ventricle becomes visible along the ventral border.
38. Upon reaching the 4^th^ ventricle, take one additional section of 400 μm thickness at a cutting rate of 60–80 μm/s.
39. Take a slow (10–50 μm/s) trim slice of 150–250 μm thickness.

***Note:*** Adjust the thickness depending on the visibility of landmark structures, such as the nucleus ambiguus.^2^

40. Verify by visual inspection that the beginning of the inferior olive is now visible in the dorsomedial region of the trimmed slice.

***Note:*** For the purposes of scRNA-seq, the goal is to obtain a “m-300 μm” slice,^3^ in which the preBötC is centered along the rostrocaudal axis of the slice.

41. Adjust the cutting blade for a final slice of 300 μm thickness and take a final slice at slow cutting speed (10–50 μm/s advance rate) (Figure 3F).

**CRITICAL:** When taking the final slice containing the preBötC, do not retract the cutting blade. It will serve as a stable surface that makes removing the slice easier.

42. Without retracting the cutting blade, gently separate the slice from the filter paper surface using a fine microknife along the dorsal border.
43. Add 1 mL of dissection ACSF to a clean 35 mm Sylgard-lined Petri dish.
44. Using a large-bore glass dropper, carefully transfer the slice from the vibratome bath into the 35 mm Petri dish, immersing it in ACSF.
45. Under 10–20× magnification of a dissection microscope, carefully punch out the preBötC using a tissue punch on the ventral border of each side of the slice.

### Dissociation of brainstem tissue punches

**Timing: 35–45 min**

This section describes the procedure for producing dissociated single-cell suspensions from brainstem tissue punches. The live cells are additionally stained with a covalently-binding viability dye (NucFix Red), so that cells that were non-viable *before* methanol fixation can be later identified during flow cytometry.

**CRITICAL:** Make sure that all dissociation glassware that will be used in this section is clean, sterile, and completely dry (see cleaning dissociation glassware).

46. Add 237 μL of Dissociation ACSF and 13 μL pronase stock to a glass conical tube (total volume 250 μL).
47. Using a ∼1000 μm I.D. (i.e., black label) fire-polished Pasteur pipette, transfer the preBötC punch-outs into the glass conical tube with the pronase-containing dissociation ACSF.

**CRITICAL:** Pronase stocks must be added to solution last, immediately prior to transferring tissue samples to the glass centrifuge tube for dissociation. Unlike more common proteases (e.g., papain), pronase is active at lower temperatures (e.g. 20°C–24°C) and will degrade itself as well as the RNase inhibitor if left standing in the incubation solution.

48. Set a countdown timer.
  a. Incubate the tissue at 20°C–24°C in the pronase-containing dissociation ACSF while gas equilibrating the solution with 95% O_2_/5% CO_2_.

***Note:*** The duration of incubation in the pronase solution increases with the postnatal age of the pups, scaling roughly linearly. We provide example timings in Table 5.

**CRITICAL:** The Dissociation ACSF solution should be continuously gassed with 95% O_2_/5% CO_2_ supplied via 1/16″ ID tubing, or a blunt syringe tip, flowing into the headspace of the glass centrifuge tubes. (i.e., there should be no direct bubbling of the solution; see Figure S1).

49. Begin tissue trituration with a large-diameter pipette (i.e., green label).
  a. Pipette up and down 10–20 times, avoiding bubbles.

**Figure 4:**
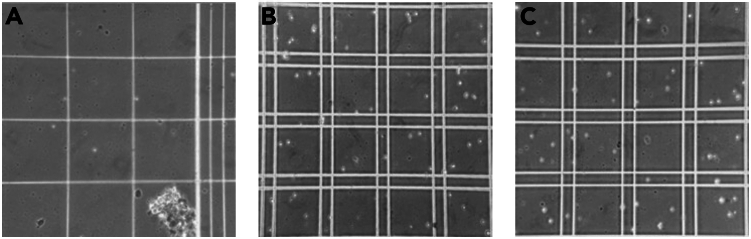
Representative images of preBötC tissue after stepwise single cell dissociation Example showing P2 preBötC triturated successively with different Pasteur pipette sizes, samples stained with trypan blue, loaded into hemocytometer and observed under microscope (10×). (A) Tissue chunks after initial preBötC trituration with the green pipette (600–800 μm). (B) Tissue chunks are then triturated with the yellow pipette (250–600 μm) to end up with smaller tissue chunks, but also triplets, doublets and single cells. (C) Cells and small tissue chunks are finally triturated with a red pipette (150–250 μm) resulting into single cell dissociation of the preBötC.

***Note:*** See Figure 4A for representative image of triturated sample at 10× magnification.

50. Repeat Step 49 two more times, using a medium-diameter pipette (i.e., yellow label; Figure 4B) and then a small-diameter pipette (i.e., red label; Figure 4C), until the tissue is completely dissociated.
51. After trituration, add 250 μL of cold storage ACSF solution to the glass centrifuge tube.
  a. Add 2 μL NucFix Red dye stock to the glass centrifuge tube.
  b. The total volume is now 502 μL.
52. Using a medium- or large-diameter Pasteur pipette, transfer the total volume from the glass centrifuge tube to a LoBind Protein 0.5 mL Eppendorf tube.
53. Fill the head space of the 0.5 mL tube with 95% O_2_/ 5% CO_2_ gas.
  a. Close the lid of the 0.5 mL tube.
  b. Incubate the cells in the solution for 5 min on ice.

***Note:*** The optimal timing for pronase incubation varies by animal age but is consistent and only needs to be determined once. We have provided example timings (Table 5), but strongly suggest users optimize these timings empirically by testing a range of incubation times and counting the resulting cells with a hemocytometer.

### Centrifugation and methanol fixation of dissociated cells

**Timing: 10 min**

**CRITICAL:** For this step you will need to prechill methanol at −20°C, and an ice bucket to keep the methanol cold while it is out of the freezer.

54. Centrifuge the cells for 5 min at 300 g and 4°C.
55. Retrieve the methanol from the −20°C freezer.
  a. Place the methanol on ice and keep it under the hood until use.
56. After centrifugation, carefully remove the supernatant until approximately 25 μL of solution remains.
57. Add 25 μL of additional gas-equilibrated cold storage ACSF to the tube.
58. Resuspend the cells by triturating 10–20 times with a small fire-polished glass Pasteur pipette.

***Optional:*** After Step 58, and before proceeding further, you may optionally use a hemocytometer to perform a cell count. In brief, stain and count cells with Trypan Blue, or an equivalent stain, according to the manufacturer’s protocol. We highly recommend including this step when troubleshooting dissociation.

59. Set the vortex to a low speed (e.g., 1–2 out of 10) and “touch” operation.
60. Hold the 0.5 mL microtube at the rim and press the conical base into the cup of the vortex.

***Note:*** The base of the tube should oscillate with the vortex, mixing the cell suspension, while the top of the tube is held steady by hand.

61. While continuing to vortex the solution, slowly add 200 μL of cold methanol (pre-chilled to –20°C) dropwise using a 200 μL pipette (approximately 25 μL/drop).

**CRITICAL:** When adding methanol, allow each drop to mix into the suspension for 1–2 seconds before adding more. Adding methanol too quickly may result in precipitates.

62. Stop vortexing.
  a. Close the lid of the tube.
63. Chill the methanol-containing cell suspension on ice for at least 15 min.

***Note:*** Methanol-fixed samples may remain on ice or in a –20°C freezer while processing additional samples.

64. Transfer finished sample tubes to a −80°C freezer for long-term storage.

**Pause point:** At this point, the protocol can be paused for up to 1 week with the methanol-fixed cells stored at −80°C.

### Rehydration of methanol fixed samples

**Timing: 50 min**

This step describes the procedure for rehydration and nuclear staining of cryopreserved cell samples for subsequent use in flow cytometry.

65. Retrieve the sample tube(s) containing methanol-fixed cells from storage at −80°C and immediately place the tubes on ice for at least 30 min.
66. Centrifuge the sample tube at 1000 g for 5 min at 4°C.
67. Remove the supernatant carefully with a 200 μL pipette tip without disturbing the cell pellet.

***Note:*** Approximately 5–10 μL of solution should be left in the bottom of the tube.

68. Add 125 μL Resuspension Buffer to the sample tube and resuspend the cells gently triturating 15–20 times with a 200 μL pipette tip.
69. Add 0.25 μL Hoechst (1:500 dilution) and gently triturate to mix the solution.
70. Incubate the sample tube on ice for 10 min in the Hoechst-containing rehydration buffer.
71. Filter cells into a new 0.5 mL Eppendorf LoBind microtube.
  a. Aspirate the entire volume from the original sample tube with a 1000 μL pipette.
  b. Filter the cells through a 40 μM Flowmi cell strainer into the new 0.5 mL LoBind microtube.
72. Use the cell suspension in the 0.5 mL LoBind microtube for subsequent cell dispensing steps via flow cytometry.

**CRITICAL:** Nuclear staining is essential for efficient cell sorting, since membrane permeabilization via methanol distorts forward and side scattering profiles during flow cytometry.

### Flow cytometry

**Timing: 10–20 min/plate**

This step details the operating parameters and procedure for dispensing rehydrated dissociated cells into 384-well PCR plates for subsequent single-cell RNA-seq library preparation.

The flow cytometer must be calibrated so that dispensing cell samples into each 384-well plate finishes in less than 30 min after filtering cells (Step 71). Flow cytometer instrument settings affect sorting efficiency and can vary between instrument models. Below we provide details and guidelines about the general gating choices that should be applicable across different sorters.

**CRITICAL:** Lysis buffer should be predispensed into 384-well plates according to the intended downstream library preparation method. Details about volume and buffer composition for SmartSeq3 (as used here) are described in the original methods paper.^4^

73. Operate the flow cytometer in single cell dispense mode with a 100 μm nozzle (disposable microfluidic chip).

***Note:*** Plate wells should be indexed according to experimental design (e.g., divide plate columns according to treatment groups).

74. Operate the sorter with both the sample tube holder and 384-well collection plate chilled to 4°C.

***Alternatives:*** We use 100 μm nozzles for dispensing cells (which are embedded in disposable microfluidic chips used with the Sony SH800S), but larger nozzles sizes may also work.

**CRITICAL:** It is strongly recommended that flow cytometer instruments be operated by trained individuals according to the manufacturer specific procedures.

**CRITICAL:** Rehydrated samples should be completely dispensed into plates (including time to sort) within 30 min after Step 72 to avoid cell clumping.

75. One sample (i.e., rehydrated cells from one mouse) is sufficient to fill 192 wells of a single 384 well plate. Two samples can be dispensed to a single plate by sequentially dispensing the samples to each half of the plate.

***Note:*** 192-wells is a conservative lower-bound on the number of cells that can be obtained from a single sample of microdissected preBötC punchouts. Dispensing to more wells is possible in samples with high cell yields or pooled samples. Dispensing into 192 wells also allows paired sample comparisons, by addressing control and treatment groups to each half of the plate.

**CRITICAL:** If cell yield is low, a single sample may be insufficient to fill 192 wells. In this case, consider pooling multiple samples into a single 0.5 mL microtube to ensure efficient use of the plate.

76. Gate cells first by blue fluorescence (Hoechst) versus back scatter area to eliminate debris and select cells with intact nuclei.
77. Eliminate cells that were non-viable prior to methanol fixation by gating for red fluorescence (NucFix Red).
78. Eliminate possible debris and large clumps of cells/debris by gating forward scatter area versus back scatter area.
79. Finally, eliminate doublets by gating with forward scatter area versus forward scatter height.

**Figure 5:**
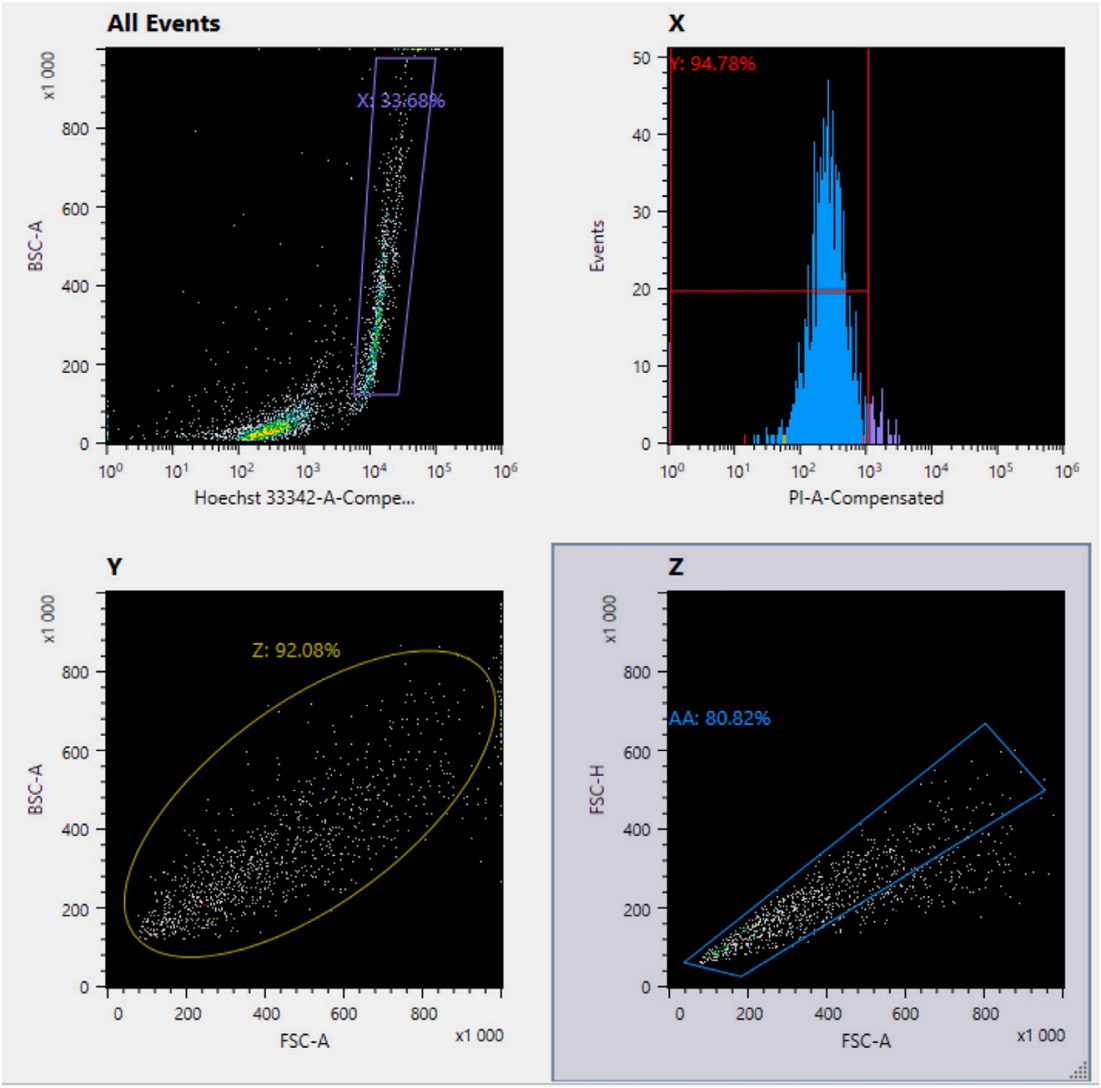
Flow cytometry gating Depiction of the gating regions and example of a high-quality sample of cells made using this protocol. Upper Left: Cells are selected by intensity of Hoechst staining versus back scatter area. Only cells with well separated Hoechst fluorescence (intact and stained nuclei) are retained. Upper Right: Cells are gated by the intensity of a red viability stain. Red lines indicate the lower and upper bounds. The horizontal red line indicates the extent of the threshold window. Lower left: Aggregates and debris are further removed by excluding outliers in back scatter area versus forward scatter area. Lower Right: Potential doublets are removed by excluding cells that skew rightward when measuring forward scatter area versus forward scatter height. The percentages are indicative, representative of a high-quality sample.

***Note:*** Examples of the selection windows and cutoffs for each of the four event gates are provided in Figure 5.

80. After dispensing cells, immediately cover the 384-well plates with foil seals.
  a. Briefly centrifuge plates at 1000 g for 1 min at 4°C.
  b. Keep the plates temporarily on dry ice until transfer to a −80°C freezer for long term storage.
81. Proceed with SmartSeq3 cDNA library preparation.^4^^,^^5^

**CRITICAL:** We initially calibrated selection windows for flow cytometry by doing a mock experiment with samples prepared as described in Steps 1–72. The resulting settings were saved as a template for reuse during subsequent sample collections. Thereafter, on each day of cell sorting, we used fluorescence and scattering measurements from the first sample tube of the day to make marginal adjustments to the template before dispensing cells into PCR plates. Specifically, the intensity of Hoechst and NucFix Red staining can vary between sample batches and must be compensated for. The density of gated cells visualized in the backscatter versus Hoechst plot (see Figure 5, upper left) will shift left or right depending on the average intensity of blue fluorescence. The enclosing polygon gate window must therefore be recentered. Similarly, changes in NucFix Red intensity require adjusting the upper threshold of the red fluorescence histogram gate (see Figure 5, upper right). These batch-specific adjustments were preserved for all subsequent samples that we sorted on the same day.

## Expected outcomes

Our cell dissociation and cryopreservation protocols are optimized for use in subsequent Smartseq3 DNA library preparation.^4^^,^^5^ For samples from P2 mouse pups, two bilateral preBötC punchouts from a single brainstem slice yield approximately 100 *live* cells per microliter (i.e., before fixation). Generally, younger mice (e.g., P0) yield more viable cells, while older mice (e.g., >P5-7) yield fewer viable cells (Table 5).

**Table 5:** Example dissociation times and number of cells obtained from preBötC punchouts

Age of mouse	Dissociation time (min)	Example cell count (per ml)
P0	20	150
P2	25	108
P3	27	99
P4	30	100
P5	30	78
P6	35	72
P8	37	103
P10	40	72

A fraction of cells is lost during cryopreservation via methanol fixation and subsequent sample rehydration.^6^ Under optimal conditions we typically recovered approximately 50% of cells after fixation and rehydration. With a single rehydrated sample, we could consistently fill at least 128 wells of PCR microplate with single cells via flow cytometry. An entire 384-well plate can be readily filled with 3–4 samples, either dispensed independently to appointed wells or after pooling into a single microtube.

Although we have not described it here, the cell suspensions generated by this protocol could be used in alternative downstream applications. For example, the dissociated cells were cryopreserved in methanol but could be fixed in paraformaldehyde for flow cytometry and imaging applications. Live or fixed cells produced with this protocol can also be used with other downstream well-based methods for single-cell RNA-seq library preparation.^7^ In addition, droplet-based cDNA library preparation methods may be suitable, since the methanol fixation approach in this protocol was originally implemented using Drop-seq.^6^ Moreover, the single cell suspension could in principle also be used for nuclear or protein isolation and quantification.

## Limitations

The success of this protocol requires practice with taking calibrated slices of the preBötC according to anatomical landmarks defined in the literature, both for accuracy and speed. Faster dissections are better, both for sample quality and for obtaining many samples within a reasonable time frame. We recommend that the protocol is carried out by a pair, with one person doing dissections and punchouts while the second person manages dissociation, staining and methanol fixation of samples. With practice, microdissection of the preBötC (i.e., Steps 1–45) can be completed in 15–20 min. The protocol is applicable for, but yet not tested on, other specific regions of interest in the brainstem (e.g., the chemosensitive parafacial ventrolateral group).

Although cell samples are methanol-fixed and cryopreserved, we found that cDNA yield in downstream transcriptomic library preparation was best when frozen samples were rehydrated and dispensed into PCR plates within 1 week of cryopreservation. Typical cDNA yields for 384 cells (one full microplate) were in the range of 8–50 nM after tagmentation, pooling, and purification.

## Troubleshooting

### Problem 1

Reduction in cell yield over repeated cell harvest across multiple weeks of experiments.

### Potential solution

• Pasteur pipettes and conical glass centrifuge tubes are coated with silicone to reduce cell loss during trituration and sample transfer (see silanization of dissociation glassware). Once silanized, the coating is relatively durable and will endure repeated usage, cleaning, and sterilization. However, we recommend that the coating be refreshed every 2–4 weeks, depending on how heavily glassware is used.
• Frozen aliquots of pronase (see preparation of 10x pronase stock) will degrade over time, resulting in poor yields after enzymatic dissociation in Steps 46–50. We suggest making a fresh batch of pronase if stocks are more than 4 weeks old.

### Problem 2

How do I measure the diameter of the tips if I don’t have a microforge?

### Potential solution

In Step 11 (fire-polishing glass Pasteur pipettes), we used a microforge with an eyepiece reticle scale to measure the inner diameter of glass pipette tips, but any benchtop microscope with a microscale can be used instead.

### Problem 3

After rehydrating cryopreserved samples, there are too few cells detected during sorting with flow cytometry.

### Potential solution

• This could result from cell loss when aspirating the supernatant after spinning down cells in Step 67. A pellet of cells may or may not be visible at the bottom of the microtube. In all cases, the supernatant should be aspirated slowly, drawing liquid from near the surface of the volume and leaving approximately 5–10 μL of volume in the bottom of the tube. Try also switching to a smaller volume pipette tip (e.g., 20 μL) to aspirate the last 50 μL of supernatant.
• This could be a symptom of poor Hoechst staining prior to sorting. Try increasing the time of incubation with the dye in Step 117 by 2–3 min.
• This could result from prolonged sample storage time following Step 64. Storage time should not exceed 1 week for best results.

### Problem 4

The volume of dissociation ACSF reduces due to evaporation during enzymatic incubation of the tissue (Step 48) and subsequent cell yields are poor.

### Potential solution

The flow of gas applied to the head space of the dissociation ACSF during Step 48 should be minimized to equilibrate the solution without rapidly evaporating the solution. In general, the gas should be applied to the headspace and not be strong enough to generate bubbles or agitate the solution.

### Problem 5

When sorting cells using a flow cytometer, it takes a long time to fill the wells with single cells. Sometimes the nozzle of the flow cytometer gets clogged.

### Potential solution

This could occur if rehydrated samples have sat for longer than 30 min before dispensing into PCR plates or if the concentration of cells is too low. Rehydrated cells will begin to clump over time and clumps can clog the instrument or be rejected as doublets, slowing down the sorting process. After filtering rehydrated cells in Step 71, samples should be immediately loaded into the flow cytometer and sorting should be finished within a maximum 30 min. If sorting is still too slow, try reducing the volume of rehydration buffer added in Step 68 (e.g., 110 μL instead of 125 μL) to increase the concentration of cells in the sample volume. If the flow cytometer runs out of volume before filling the wells of the plate, consider pooling two or more samples into a single 0.5 mL microtube. Do this by performing Steps 65–70 for each sample independently. At Step 71, filter all samples into the same 0.5 mL microtube for subsequent sorting.

## Resource availability

### Lead contact

Further information and requests for resources and reagents should be directed to Eric Herlenius (eric.herlenius@ki.se).

### Technical contact

Naify.ramadan@ki.se

### Materials availability

The necessary reagents for this protocol are all commercially available.

### Data and code availability

The development of this protocol did not generate or analyze datasets.
