# Supplementary material for: Protocol to dissect and dissociate the mouse brainstem for single-cell RNA-seq applications
Source: STAR Protoc. 2024 Mar 8;5(1):102908. doi: 10.1016/j.xpro.2024.102908 (PMC10940983; doi:10.1016/j.xpro.2024.102908)

Supplementary Figure 1: Glass centrifuge tubes and gas manifold for tissue dissociation, related to Step 48.

Silanized glass centrifuge tubes are used for tissue dissociation. To maintain pH of the dissociation ACSF, the vials are continuously gassed with 95% O_2 – 5% CO_2. The gas flows into the headspace of the tubes without directly bubbling the solution. One-hole rubber stoppers are used to seal the tubes, minimizing excessive gas use. On the right, a custom-made, hand-built manifold used for turning individual gas lines on and off.

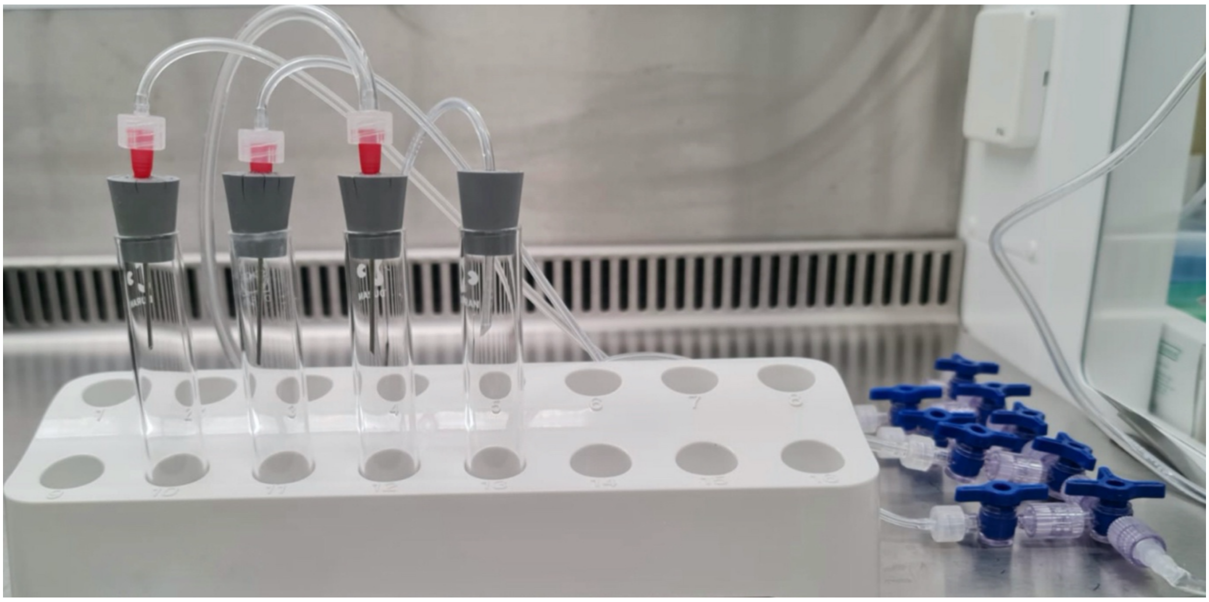

Supplement: Document S1. Figure S1 [file mmc1.pdf]
